# Supplementary material for: Association of Ventilatory Disorders with Respiratory Symptoms, Physical Activity, and Quality of Life in Subjects with Prior Tuberculosis: A National Database Study in Korea
Source: J Pers Med. 2021 Jul 19;11(7):678. doi: 10.3390/jpm11070678 (PMC8305056; doi:10.3390/jpm11070678)
Supplement: Supplementary file 1 [file jpm-11-00678-s001.zip › jpm-1273466-supplementary.pdf]

Supplemental Table S1. The impact of ventilatory disorder on respiratory symptoms, physical activity limitation and EQ-5D index value in subjects with prior TB

	Model	Normal (n = 1,466)	Ventilatory disorders	
			Obstructive ventilatory disorders (n = 783)	Restrictive ventilatory disorders (n = 420)
Respiratory symptoms	Crude model	Reference	2.09 (1.37, 3.21)	1.61 (0.95, 2.72)
	Adjusted model*	Reference	1.63 (1.05, 2.82)	1.57 (0.97, 2.26)
Cough	Crude model	Reference	2.30 (1.27, 4.17)	0.95 (0.43, 4.17)
	Adjusted model*	Reference	1.68 (0.80, 3.53)	0.68 (0.26, 2.71)
Sputum	Crude model	Reference	2.13 (1.34, 3.39)	1.70 (0.93, 3.12)
	Adjusted model*	Reference	1.85 (1.03, 3.37)	2.01 (0.99, 3.03)
Dyspnea	Crude model	Reference	4.05 (1.30, 12.62)	2.49 (0.63, 9.81)
	Adjusted model*	Reference	4.19 (1.03, 17.14)	3.03 (0.62, 14.76)
Physical activity limitation	Crude model	Reference	7.20 (2.93, 17.66)	2.98 (0.76, 9.45)
	Adjusted model*	Reference	6.59 (1.98, 21.93)	2.73 (0.73, 10.24)
EQ-5D index	Crude model	Reference	- 0.02 (- 0.04, - 0.07)	- 0.03 (- 0.05, - 0.06)
	Adjusted model*	Reference	- 0.001 (- 0.02, 0.02)	- 0.03 (- 0.05, 0.001)

Data are presented as a ratio (95% confidence interval) or a difference estimate (95% confidence interval). * Adjusted for age, sex, body mass index, smoking amount (pack-years), education (categorized as >high school or ≤high school), and family income (categorized as low or high).
EQ-5D, EuroQoL five dimensions; TB, tuberculosis.
